# Athletic Trainer’s Varying Levels of Awareness and Use of Disablement Model Frameworks: A Qualitative Study

**DOI:** 10.3390/ijerph20054440

**Published:** 2023-03-02

**Authors:** Rylee T. Haffey, Matthew J. Rivera, Justin P. Young, Zachary K. Winkelmann, Lindsey E. Eberman

**Affiliations:** 1 Department of Applied Medicine and Rehabilitation, Indiana State University, Terre Haute, IN 47809, USA; 2 Department of Exercise Science, University of South Carolina, Columbia, SC 29208, USA

**Keywords:** athletic trainers, disablement models, patient-centered care, environmental factors, societal factors

## Abstract

In healthcare, disablement model frameworks aim to improve the delivery of patient-centered care through the recognition of patient factors beyond impairments, restrictions, and limitations, which include personal, environmental, and societal factors. Such benefits translate directly to athletic healthcare providing a mechanism for athletic trainers (ATs), as well as other healthcare professionals, to ensure that all aspects of the patient are managed prior to returning to work or sport. The purpose of this study was to investigate ATs recognition and use of disablement frameworks in current clinical practice. We used criterion sampling to identify ATs who were currently practicing from a random sample of ATs that participated in a related cross-sectional survey. A total of 13 participants engaged in an online, audio-only, semi-structured interview that was audio-recorded and transcribed verbatim. A consensual qualitative research (CQR) approach was used to analyze the data. A coding team of three individuals used a multi-phase process to construct a consensus codebook that identified common domains and categories among the participants’ responses. Four domains emerged regarding ATs’ experiences and recognition of disablement model frameworks. The first three domains were related to the application of disablement model frameworks: (1) patient-centered care, (2) limitations and impairments, and (3) environment and support. Participants described varying degrees of competence and consciousness regarding these domains. The fourth domain related to participants’ exposure to disablement model frameworks through formal or informal experiences. Findings suggest that ATs largely demonstrate unconscious incompetence regarding the use of disablement frameworks in clinical practice.

## 1. Introduction

Athletic trainers (ATs) are highly qualified, multi-skilled healthcare professionals who provide service or treatment under the direction of or in collaboration with a physician [1,2]. ATs combine their formal education and training with state’s statutes, rules, and regulations to provide healthcare services [1,2]. These services include primary care, injury and illness prevention, wellness promotion and education, emergent care, examination and clinical diagnosis, therapeutic intervention, and rehabilitation of injuries and medical conditions [1,2]. The athletic training profession contributes to public health through integrating prevention programming with annual screenings and management of illnesses, aligning with primary, secondary, and tertiary considerations to promote positive patient outcomes [3,4]. For example, within sport-related concussions (SRC), ATs acknowledge the differences in SRC reporting behaviors associated with return-to-activity timelines. ATs aim to mitigate the risks of SRC through intake of extensive patient history and education, as well as promotion of body awareness, to in turn, ensure safe sport participation [5,6]. As defined by the American Public Health Association, “public health promotes and protects the health of people and the communities where they live, learn, work, and play” through injury and illness prevention and wellness promotion through the practicing of healthy behaviors [3]. Additionally, ATs actively contribute to injury surveillance and data collection, all consistent with public health initiatives [3].

Patient-centered care is a critical aspect of delivering quality healthcare by improving healthcare safety and health-related quality of life. Functional limitations and restrictions during activities of daily living, in addition to mental and physical demands, create various detrimental impacts that must be considered. [7,8]. Patient-centered care aims to achieve improved health outcomes and patient experiences, greater patient satisfaction, and chronic disease management [7,8]. Integration of patient values into plans of care in each phase of their medical consultation, treatment, and follow up is a priority in patient-centered care to best drive patient-provider relationships and enable providers to prioritize the patient’s voice and perspective [8,9]. Consistent with public health initiatives, athletic medicine aims to promote the recognition of implicit causes, such as inequalities and social determinants of health that may impact the patient’s likelihood of reaching optimal recovery. Acknowledging such causes that may impact a patient’s well-being, healthcare professionals must work together to reduce disparities seen within the American healthcare system, especially within access to care [9,10].

Despite growing conversation about patient-centered care and its impact on well-being, healthcare remains largely disease-driven with patients lacking complete autonomy in their care [10,11,12]. To best demonstrate valuable and comprehensive strategies to promote a shift from traditional, provider-driven approaches, psychosocial frameworks such as The Disablement Model and International Classification of Functioning (ICF) Checklist can serve as appropriate tools for the description of health and health-related states [13,14]. Disablement models and the ICF are conceptual frameworks for clinical practice that organize clinical decision-making and documentation to comprehensively address the interaction between a person’s health condition, personal factors, and environmental factors combined [14]. Implementing such frameworks facilitates these principles by encompassing patients’ experiences and the clinicians’ objective measures, while creating a common language and consistency for interprofessional care teams to rely upon and concisely measure the effectiveness of clinical decision making, therapeutic interventions, and most importantly, patient preferences [4,8,13]. For example, one study found a significantly higher use of patient-rated outcomes (PRO) when using ICF-based patient assessments [15], where another describes from a nursing perspective, feasibility and relevance of ICF application in clinical practice [16]. With the previous data and positive patient outcomes and feasibility in practice, the ICF and disablement model are strategies for improving a patient-centered approach in clinical practice [15,16]. The benefits of patient-centered care and disablement model frameworks are only as strong as the integration by ATs; however, current data does not reflect to what level ATs know or are using these mechanisms.

Unconscious or implicit bias is a characteristic in every human being and healthcare providers are not spared from this psychosocial response. The delivery of care and patient outcomes can often be influenced by unconscious bias, defined as the associations or attitudes that reflexively alter our perceptions, thereby affecting behavior, interactions, and decision-making [17]. With this in mind, often, certain structural inequities are being ignored in healthcare [9,10]. The social and relational levels of disablement models guide clinicians beyond physical parameters. They attempt to account for disability from a holistic perspective that involves a person’s inability to fulfill an expected societal role, which impacts the patients’ health-related quality of life over time [7,12,18]. The intersection between athletic medicine and public health efforts is at the point of improving health equity by delivering comprehensive patient-centered care [3]. The use of disablement frameworks promotes uniformity of patient information across clinical environments and should be deemed essential for positive clinical outcomes assessments and collection of patient-rated evidence, which is the most valuable form of outcomes evidence [13].

To date, the current literature provides extensive information regarding the components and potential benefits of disablement model frameworks in clinical practice such as The Disablement Model and World Health Organization International Classification of Functioning (WHO ICF) [3,13,18,19]. For example, the use of disablement models will allow the athletic training profession to communicate, measure, and prioritize the healthcare needs of patients [13,14]. Recommendations have been tailored to carry out research about the applicability of these models to athletic training practice [18]. To our knowledge, studies demonstrating the lived experiences of ATs integrating disablement model frameworks is lacking. Awareness towards patient-driven care and motivation for consistent use of disablement model frameworks is crucial to the profession of athletic training. Therefore, the purpose of this study was to investigate ATs’ recognition and use of disablement frameworks in current clinical practice.

## 2. Materials and Methods

### 2.1. Study Design

We used a phenomenological approach with consensual qualitative research (CQR) analysis methods to investigate ATs experience and recognition of psychosocial frameworks, like disablement models, to assess the competence of whole-person healthcare considerations. This approach has been used in previous investigations in athletic training. We adhered to the Standards for Reporting Qualitative Research to guide the reporting of the study [20]. The research was deemed exempt by the Indiana State University Institutional Review Board.

### 2.2. Participants

A total of 13 interviews were completed in this study (Table 1). More than half of the participants received a post-professional master’s degree (*n* = 8). Participants mainly worked within the college and university setting (*n* = 8), with the remaining participants working in the secondary school setting (*n* = 4) and in the health/fitness/sports performance field (*n* = 1). On average, participants were 36 ± 12.12 years old and had 11 ± 11.74 years of experience.

### 2.3. Interview Protocol

As no interview protocol existed specific to the purpose, two members of the research team created a list of questions to explore ATs’ lived experiences using disablement model frameworks and translation of information into clinical practice. The initial interview protocol was then sent to, and reviewed by, four ATs who are trained in qualitative research and who have studied disablement model frameworks. Once the interview protocol was finalized based on the feedback provided, the interview was piloted with a small group of ATs that were clinically practicing but were not included in the final data collection or analysis. The pilot interviews allowed the research team to ensure that the question sequence flowed logically, determine an average time to complete the interview, and practice with the semi-structured nature for follow-up questions that could arise. No changes were made to the interview protocol after the pilot interviews were concluded and a final set of nine questions was used for data collection found in Table 2.

### 2.4. Procedures

The initial recruitment email was sent to ATs who participated in a cross-sectional study on knowledge, familiarity, and use of disablement frameworks. Within the previous study, the survey asked participants if they were willing to complete a follow-up interview to reflect on their current exposure to psychosocial frameworks and integration into clinical practice. We contacted those who answered yes and indicated in the survey that they were currently practicing as an AT. The recruitment email included a brief study description, an online link (Qualtrics©, Provo, UT, USA) to the informed consent document, and a brief demographics survey. The participants were able to review the online informed consent document and provide their contact information. Once a participant indicated their willingness to participate and complete the demographic survey, the primary investigator contacted them to schedule an interview time. At the scheduled time, the participant joined the primary investigator on the audio platform (Zoom Video Communications©, Inc., San Jose, CA, USA) to conduct the interview. Once verbal consent was obtained, the primary investigator began the audio recording and conducted the interview. On average, the interview duration was 15 min. At the point of data saturation, or the point at which no new data was emerging from the interviews, transcripts from each interview were transcribed verbatim, de-identified, and checked for accuracy by the primary investigator before being sent to the participant for member checking. Member checking is the process where a participant ensures accuracy of the recorded statements and is used to establish trustworthiness of the data. Interview transcripts were returned via email to each participant and they were asked to review their transcript to ensure that their responses best captured and appropriately reflected their lived experiences. No content changes were requested by the participants during member checking.

### 2.5. Data Analysis and Trustworthiness

A panel of three researchers engaged in the CQR method of analysis. During phase one, the research team reviewed four transcripts to identify domains reflected within the data. The team met to compare core ideas that emerged from the data and develop an initial codebook of the domains. In phase two, the initial codebook was independently applied by each team member to two transcripts used in phase one, then two new transcripts. The team then met to discuss findings and ensure continued reflectiveness of the data within the codebook. Transitioning to phase three, the team applied the codebook to the remaining seven transcripts. After each transcript was coded, an internal audit was completed to ensure that each code was reviewed by two members of the research team. Differences were discussed until consensus was reached. A cross-analysis was then conducted by the primary investigator to assess that each domain appropriately reflected participant responses. Next, an external reviewer was sent a series of four coded transcripts to finalize the consensus codebook. No major revisions were applied to the codebook during the external review. At the conclusion of the cross-analysis and external review process, a frequency calculation of the categories was performed. Categories were assigned to one of four frequency classifications: “general” meaning the domain was identified in all or all but one (*n* = 12–13) transcripts, “typical” meaning the domain was identified in (*n* = 6–11) transcripts, “variant” meaning the domain was identified in (*n* = 2–5) transcripts, “rare” if in one transcript, and “none” if not identified in any participant transcript [21]. To conclude the analysis, a series of direct quotes were selected to further support the specified domains established. Credibility and trustworthiness were achieved by member checking, triangulation, and internal auditing.

## 3. Results

### 3.1. Domains

Four domains emerged regarding ATs’ experiences and recognition of disablement model frameworks (Table 3). The first three domains were related to the application of disablement model frameworks: (1) patient-centered care, (2) limitations and impairments, and (3) environment and support. Participants described varying degrees of consciousness regarding these domains including: expressed desire, conscious competence, conscious incompetence, and unconscious incompetence. The fourth domain related to participants exposure to disablement model frameworks through formal or informal experiences.

When evaluating the data, a pattern regarding participant lived experiences emerged similar to the competence hierarchy. The competence hierarchy is a framework for achieving mastery and gauging one’s mindset towards accomplishing a task [22]. We characterized the data using the competence hierarchy, whereby conscious competence was demonstrated when participants accurately acknowledged or implemented a domain related to disablement model framework use. Participants displayed conscious incompetence when they actively acknowledged that they did not know or did not actively use the domain. Unconscious incompetence was demonstrated when participants expressed that they knew about or implemented a component of the disablement models but were not accurately applying the concepts. Expressed desire, although not part of the competence hierarchy, represents data when participants spoke about a desire towards change and integration of disablement model framework components into clinical practice.

### 3.2. Patient-Centered Care

In this category, participants described actions, attitudes, and behaviors that aligned with patient-centered care such as shared decision making, setting patient goals, and consideration of external factors that impact a patient’s response to injury. This was the most frequently described domain.

#### 3.2.1. Expressed Desire

Participants typically described wanting to be more patient-centered in their approach to care. For instance, when asked how the disablement model framework fits in their mindset of patient-centered care, participants demonstrated a desire to actively apply a patient-first approach. For example, Participant 8 described the strong desire to shift their mindset to be patient-centered and tailor decision-making in their care towards patient goals.

*“I think this [disablement model] is something that I did not previously have much experience with. In the last two years or so, it is something that is coming farther and farther into the forefront of my mind in terms of the decision-making process with patients. It fits into my mindset, by allowing me to identify problems or things that are inhibiting them from being completely healthy. It helps us identify goals that they’re trying to reach and essentially helps us identify outcomes that we as providers want to reach throughout the post injury or, any sort of similar situation.”*

#### 3.2.2. Conscious Competence

Typically, participants were consciously competent when describing principles of patient-centered care, although many participants failed to associate disablement model frameworks with an active mindset. Participants who spoke to an active mindset acknowledged the importance of routinely identifying holistic aspects across patient encounters. Specifically, Participant 9 described a heightened awareness where such frameworks guided their approach to patient-centered care.

*“[A disablement model framework] is not something where I’m consciously writing down or making some sort of map or listing certain aspects of it. It’s more something that stays in the back of my mind when I’m considering a patient’s well-being or their plan of care.”*

#### 3.2.3. Conscious Incompetence

Many participants described having minimal knowledge of the terminology and components of disablement model frameworks, while acknowledging the potential use of these frameworks based on their current behaviors, regardless of the structure or organization that disablement model frameworks can provide. Participant 1 highlighted this when asked to relate barriers of implementation in their clinical practice; they described lacking awareness and relying on their current personal clinical expertise and experiences to deliver patient-centered care.

*“I’m not completely sure what’s entailed in the model, the framework. I am not sure exactly what barriers I come across. I am sure I have, and probably use the model without knowing it.”*

#### 3.2.4. Unconscious Incompetence

A handful of participants lacked awareness towards the use and appropriate application of disablement model frameworks. Various participants provided insight misaligning the goal of disablement models with the intended outcomes. Participant 5, for example, inaccurately described the relevance of frameworks in clinical practice and guiding patient-centered care.

*“[The disablement model] is useful, it is good to implement it, so that way you can show professionalism.”*

### 3.3. Limitations and Impairments

Typically, participants described experiences in addressing the physical condition of a patient, alluding that the limitation and impairments that are present in an injury are more frequently considered in comparison to external factors within disablement frameworks.

#### 3.3.1. Expressed Desire

Interestingly, when asked to describe how they integrate the impacts of impairment, limitation, and restriction of injury into the care provided, only two participants highlighted an explicit desire to create a patient-driven approach which integrates limitations, modifications, and goal setting in activities of daily living. For example, Participant 5 described specific aspects that consider the whole patient, demonstrating a strong desire to gain awareness and exposure to disablement model frameworks.

*“Each case is specific to each individual with their own goals and their own activities of daily living in mind; I know that.”*

#### 3.3.2. Conscious Competence

Nearly all participants shared how they integrate the patients’ limitations and impairments into their decision-making. Identifying these factors was particularly valued by these participants, creating the foundation of rehabilitation progression and selecting evidence-based interventions to treat each impairment of the injury. Participant 6 comprehensively addressed clinical signs, functional limitations, and disabilities that impact both personal and societal roles of the patient in terms of limitations and restrictions.

*“Using limitations, in terms of what are their limitations in performing a specific task? Then limit activity as you discharge the patient from an injury. In terms of restrictions, what are they able to participate in, not just sport related but also school and life related.”*

#### 3.3.3. Unconscious Incompetence

Participants oftentimes misidentified limitations and restrictions beyond the definition of what the disablement model describes. For instance, participants used changes in societal role to describe this level of the disablement model without acknowledging true activity limitations and restrictions. For example, Participant 2 unsoundly described the integration of limitations and impairments exclusively focusing on aspects of patient participation.

*“You’re taking them away from something that they’ve done for a lot of years and, especially, working in a college setting, they’ve been doing it for a long time at a very high level. So, just being there and letting the athlete or patient know that I’m here for you, we can set you up with whatever you need, whenever you need it.”*

### 3.4. Environment and Support

When describing the environmental and support factors for patient cases, participants were regularly able to accurately describe what might be considered, though their examples and clinical application did not often align with the defined categories.

#### 3.4.1. Expressed Desire

Despite ATs demonstrating conscious competence within patient-centered care, a few participants described barriers to adopting the use of disablement model frameworks relating to staff buy-in and resistance to change in current clinical settings. The desire for surrounding colleagues to be open-minded in the use of such frameworks to consider environmental factors, support factors, and holistic care was apparent. Participant 9 spoke of firsthand encounters of attempting to guide ATs to use disablement model frameworks, although their years of experience impacted their ability to shift a change of behavior in senior roles.

*“When I was lower ranked I faced difficulty with my colleagues and coworkers around me, trying to help them understand that there is more than just the physical aspect going on and there’s other factors that affect a patient in their treatment.”*

#### 3.4.2. Conscious Competence

Generally, most participants considered the influence of some environmental and societal factors on disability, anticipating potential changes between the patient and their environment. A patient’s means of transportation, family support, and role on their sports team were commonly addressed throughout the plan of care, although many failed to identify such factors comprehensively and consistently. Participant 13 emphatically reflected on various scenarios, highlighting the importance of addressing environmental and societal factors impacting the patient’s injury recovery and outcome.

*“It is the wintertime, and someone has a lower extremity injury. You would have concern about how they get to their classes, how they get to the athletic training room? Do they need crutches? Do they need additional support to get to where they need to go? So, understanding the environment is important in gathering if they have limitations with transportation.”*

#### 3.4.3. Conscious Incompetence

Four participants demonstrated conscious incompetence, or a self-awareness of their shortcomings, regarding gathering information about a patient’s environmental and societal impacts. Commonly, these participants acknowledge that they attempt to consider factors like living conditions while not taking the opportunity to efficiently expand on the impacts. For instance, Participant 4 broadly described a societal impact that does not capture the extensive considerations that need to be addressed, as well as acknowledging that these factors are not conformed across all patients in their population.

*“There are definitely other patient factors that I’m unaware of, so I try to ask some patients if they are in a third-floor apartment, or if in their dorm are there elevators? I do try and take those things into account.”*

#### 3.4.4. Unconscious Incompetence

More than half of the participants did not appropriately identify environmental and support factors when considering integral aspects of patient-centered care. Participants described factors that did not relate to the definition or expected considerations that would identify as an environmental or societal concern. Participant 12, for example, demonstrated a lack of awareness of how to appropriately inquire about environmental factors that may impact a patient’s injury progression by sharing, “Considering housing conditions? I do not ask such information, because I usually just see them during school hours.”

### 3.5. Exposure

Less than half of the participants received formal education on disablement model frameworks with minimal associated clinical application, while the remaining participants acknowledged that they informally and independently sought out available evidence on frameworks in their professional career.

#### 3.5.1. Formal

Despite five participants having previous formal exposure to such frameworks throughout their professional programs, only one participant actively applied the disablement model mindset in response to their formal exposure. It was common for participants to need a refresh; to remind them of how to appropriately apply disablement model frameworks into clinical practice. It was apparent even with formal exposure that ATs were not actively adopting the holistic mindset guided by disablement model frameworks. For example, Participant 4 shared a personal experience in their professional program where they did not prioritize clinical reinforcement and application for efficient preparedness upon entry-level practice.

*“I remember studying [disablement model frameworks] in college and I remember it getting brought up here and there and touching on points on the disablement model, but it’s never been a forefront of my mind, whereas I feel like it should be, because it was a big deal in college, it was eye opening.”*

#### 3.5.2. Informal

Most of the participants desired improvement in their current experiences in using disablement model frameworks, expressing accountability in taking the time to refer to the available literature and potential continuing education opportunities. When asked to provide further insight on the use of disablement model frameworks in the practice of athletic training, Participant 11, for example, described needing to bridge the gap in athletic training research, to translate more clinically, supporting our notion that informal exposure may not be enough to promote the aptitude of disablement models in routine clinical practice. ATs relying on informal exposure may not be able to fairly embrace such frameworks to their fullest potential, especially ATs who have clinically practiced prior to the change in Commission on Accreditation of Athletic Training Education standards. Participant 12 identified and reflected on their knowledge gaps, describing their planned self-improvement:

*“I’m going to research the disablement model again, and see what it is, what is included in it, and then try to work in where I feel like I’m lacking because there’s always room for improvement.”*

## 4. Discussion

### 4.1. Competence Model

The stages of the competence model uses a sequential model to demonstrate the increasing competence of learners [23]. ATs in this study largely demonstrated unconscious incompetence in regard to appropriately identifying characteristics included in the disablement model framework categories. The terminology consciousness and competence can be related to awareness and skill level, respectively. According to the competence model, the first stage, or unconscious incompetence, an individual lacks self-awareness towards recognizing the skill or knowledge deficit [23]. The individual may deny the usefulness of the skill and does not understand how to use or apply the skill. Consistent with our findings, various participants misaligned the goal of disablement models, often unaware of the intended goals, benefits, and direct clinical application. Within conscious incompetence, stage two, an individual is able to recognize the skill or knowledge deficit, having the self-awareness of not understanding how to use or apply the skill [23]. The individual may find value and motivation in addressing the shortcoming but may not recognize the knowledge or skill gap to execute. In this study, ATs demonstrated variant levels of conscious incompetence, demonstrating a further knowledge gap of disablement models in athletic training. A previous study examined family medicine residents’ performances during a standardized patient experience [24]. Researchers found that participants who performed within the top third percentile were able to improve their self-assessments to be more accurate when presented with benchmark videos [24], therefore equipped to apply immediate changes to their clinical practice following exposure to observation of others’ behaviors and make the move from unconscious to conscious thinking. The next stage is conscious competence; an individual actively uses the skill and understands how to use or apply the skill where conscious thought is necessary. The final domain, unconscious competence, describes when an individual has extensive exposure to a skill, and it can be performed easily, likely executing another task simultaneously. Unconscious competence was not included in this data set as this level of consciousness required direct observation of skill implementation. Rather, the domain of expressed desire emerged, which encompassed an expressed aspiration towards acquiring a specific skill and consciously thinking about how to initiate such change in themselves.

### 4.2. Readiness for Change

Expressed desire to improve the approach to patient-centered care using disablement models was evident within the study population, where almost all participants provided a response to integrate a more patient-centered approach. The idea of change and how we evolve personally and professionally can be challenging, particularly for those well established in their practice or institutions [18]. Yet, the adoption of patient-centered care and use of disablement model frameworks in practice has the potential to positively impact patient outcomes. For example, a previous study supports practicing within an interprofessional team as an important piece in the ICF and disablement model frameworks and is generally associated with better outcomes by achieving full understanding of the patient [16]. The inability of ATs to appropriately identify and provide examples for how they integrate patient-centered care raises the concern that even though ready to be patient-centered, ATs lack the skills to do so comprehensively. Resonating with the long-standing transtheoretical model, changing a behavior is not a coincidence, rather a process where individuals are in different stages of readiness or desire for change [25]. The five stages progress through precontemplation, contemplation, preparation, action, and maintenance [25,26]. In this model, certain factors can impact the progression between each stage and the time necessary for change including decisional balance and self-efficiency [25]. When relating the stages of change and competence, unconscious incompetence algins with the pre-contemplation phase where various ATs who participated were often unaware that their behavior towards disablement model frameworks was potentially problematic for their clinical practice. Otherwise, ATs that are in a stage of self-reflection and self-awareness, currently attempting to initiate change personally or within their system, align with contemplation to action. Experiencing expressed desire is the first step in behavior change and is adequate for ATs to identify the areas for potential improvement of further use and integration of disablement model frameworks. Using the disablement model frameworks can provide a tangible mindset shift towards implementing a more patient-centered approach.

### 4.3. Disablement Model Frameworks

While providing compelling organization for patient information through a conceptual structure, disablement model frameworks independently prioritize healthcare interventions on the unique needs of each patient [12,13]. The central focus of the disablement model is to capture the patient perspective of their injury, considering each factor beyond the physical impairment where particular health constructs such as personal and environmental factors can be further evaluated [27]. Previous literature demonstrates that physical disability is more commonly focused on and considered during patient encounters in comparison to the rest of the patient’s life [18,28]. Similarly, participants in our study demonstrated higher conscious competence and no conscious incompetence with limitations and impairments that patients may be facing. The focus on physical impairments underlines the importance of ATs needing to increase self-awareness of advanced practice behaviors to focus on the whole patient. Guidance from disablement model frameworks for example, supports integration of patient-rated outcome (PRO) measures which are aimed more at assessing the health-related quality of life of the patient, determining what is important to the patient, and giving the patient a voice in their care [13]. Further, PRO measures are conceptually based on the framework of disablement models [13]. Connecting efficiency of documentation, such frameworks can help guide ATs’ current perceived barriers including time, expectations, priority, and reimbursement incentives [29]. Combining such considerations has the power to elevate the standard of patient-centered care but can only elevate through consistent action and expressed desire for change.

### 4.4. Resolving the Knowledge Gap

ATs in this study being typically unconsciously incompetent was alarming, as ATs are highly capable clinicians trained to manage other aspects of patient care, not just orthopedic conditions alone [1,2]. A lack of exposure and knowledge of addressing the whole patient may be the cause of this focus on the impairments rather than the whole patient. The Commission on Accreditation of Athletic Training Education requires the instruction of disablement model frameworks to guide the delivery and communication of patient care [30]. Standard 60 does not explain the expected application in coursework, which can influence the delivery, depth, and reinforcement that athletic training master’s students are receiving throughout their educational experiences. Our participants who had formal exposure were consciously incompetent, demonstrating an awareness that disablement model frameworks exist, but may have deprioritized its use in clinical practice due to potential limitations in the integration of these frameworks in didactic or clinical experiences. Participants acknowledged a lack of required application, expressing desire for further exposure in their professional programs and/or clinical experiences. Both formal and informal exposure combined is needed for ATs to move from an unconscious to conscious mindset of using such frameworks to guide their patient-centered care.

To improve the use of disablement model frameworks, we propose recommendations based on demonstrated consciousness and skill-level to move to the proceeding stages towards change. For example, the unconsciously incompetent learner may prioritize building awareness of purpose and the role of disablement model frameworks; first, identifying the skills needed, then engaging in observed clinical practice and standardized simulations regarding application of frameworks. The observation of practice or simulation will allow learners to have a specific identification of where they can integrate more disablement model frameworks in practice. Although they should be least prioritized during this stage as a previous study by Dunning and Kruger found that incompetent individuals fail to understand their own incompetence by solely observing the behavior of others [31]. Further, chart audits may also provide value during this stage to promote where ATs can integrate a more holistic approach to patient care through documentation. Previous literature suggests that if ATs lack overall exposure to such frameworks, they cannot be expected to apply them as a method to determine clinical practice decisions and promote evidence-based medicine [28]. This is important for athletic training educators to reflect on current curriculum standards of applying the disablement model during didactic experiences. Educators may tailor standardized patients to application of frameworks in diverse patient cases or require assigned documentation to be inclusive of each component that should be addressed within the disablement model.

ATs who acknowledge passive understanding from previous exposure, or conscious incompetence, need to improve their ability to translate elements of the frameworks into their daily interactions with patients. We suggest engaging in informal exposure including available research, regular and intentional practice reflection, and the implementation of small-scale, continuous quality improvement efforts. Integrating small changes or improvements in short cycles every 1–2 weeks can help adapt ATs’ personal needs and working environment [32,33]. As mentioned, expressed desire can drive positive experiences through self-awareness and self-reflection. Integrating professional development plans and continuous quality improvement practices including affinity diagrams and change cycles such as the plan-do-study-act (PDSA) cycles [32,33] should be prioritized. In creating a professional development plan, ATs may consider writing “SMART” goals with benchmarks, action strategies, resources, desired outcomes, and measurement of results that are related to better use of the disablement models [34]. Second, the AT should create and follow mini habits, setting benchmarks and outcomes for each [35]. Mini habits may include reading for 10 min a day or creating reminders that trigger a task to be completed. Habits are likely to persist even after conscious motivation or interest dissipates [36].

For ATs that acknowledge their formal and informal exposures to be adequate, or routine integration of disablement frameworks, they should reinforce their knowledge through teaching others by encouraging peers or colleagues to observe practice and ask questions to best share how the disablement model aids the approach to patient-centered care. The AT may also create and develop a standardized case with the help of specialists to share with ATs within the working environment or those demonstrating incompetence.

### 4.5. Limitations and Future Research

This study contributes novel evidence of ATs’ varying levels of awareness and use of disablement model frameworks, as the current literature does not reflect the experiences of ATs, instead defining the profound nature and intended use of such frameworks independently. In addition, the consistency of the code book allowed the research team to identify the domains and subdomains across participant transcripts. However, the findings of this study resulted in various limitations and therefore, future research is recommended. Future research should focus on the impact of educational interventions on behavior change in alignment with the best practices in patient-centered care. The qualitative design of this study including a small sample may not represent the experiences of all ATs and their use of disablement model frameworks in clinical practice. Less than 10% of ATs included in the previous study agreed to participate in the follow-up interview included in this study. Participants were recruited from NATA and were part of a previous study; therefore, these experiences do not reflect ATs that are not enrolled as a member of NATA. In addition, remote interviewing via online conferencing may have deterred participants with low digital literacy.

## 5. Conclusions

Disablement model frameworks have the potential to provide ATs with a comprehensive approach to improving the delivery of patient-centered care. Findings suggest that ATs included in the study largely demonstrate unconscious incompetence regarding the use and recognition of disablement frameworks in clinical practice. Due to the comprehensive nature of disablement model frameworks and usefulness in clinical practice, increased awareness of such frameworks should be considered. Moreover, implementation research regarding the effective application of disablement model frameworks is needed when considering such frameworks becoming the clinical standard in athletic training since the National Athletic Trainers Association’s (NATA) adoption in 2015 [37].

## Figures and Tables

**Table 1:** Participant demographic (*n* = 13).

Participants	Gender	Level of Education	Work Setting	Years of Experience
Participant 1	Female	Master’s	College/University	5
Participant 2	Female	Master’s	College/University	4
Participant 3	Male	Post-Professional Master’s	Secondary School	47
Participant 4	Female	Post-Professional Master’s	College/University	17
Participant 5	Male	Master’s	College/University	7
Participant 6	Female	Post-Professional Master’s	Secondary School	9
Participant 7	Female	Post-Professional Master’s	College/University	17
Participant 8	Female	Post-Professional Master’s	College/University	5
Participant 9	Female	Post-Professional Master’s	College/University	5
Participant 10	Female	Master’s	Secondary School	3
Participant 11	Male	Post-Professional Master’s	Health/Fitness/ Sports Performance	11
Participant 12	Male	Bachelor’s	Secondary School	5
Participant 13	Male	Post-Professional Master’s	College/University	8

**Table 2:** Interview Protocol.

What does patient-centered care mean to you? How does the disablement model framework, if at all, fit into your mindset of patient-centered care? If described, but not addressed: Specifically, how does the disablement model fit into how you make decisions about patient plans of care? If not described: What, if anything, do you know about the disablement model framework?
2. Generally, how, if at all, do you implement the disablement framework into your clinical practice?
3. How, if at all do you integrate the impact of impairment, limitation, and restriction of an injury into the care you provide?
4. How, if at all, do you integrate the environmental or societal impacts of injury into the care you provide?
5. What barriers, if any, have you experienced in implementing the disablement model framework into your clinical practice? What strategies, if any, have worked to help you overcome those barriers?
6. In what ways, if any, do you believe the use of the disablement framework can further establish a common language within interprofessional practice, particularly for those working within orthopedics and healthcare for the physically active? In your experience, if any, has using disablement frameworks within interprofessional encounters impacted patient-centered care?
7. In your mindset, how, if at all, can the disablement model improve patient-centered care? If described, but not addressed: Specifically, how does the disablement model improve the deliverance of patient-education in your clinical practice? If not described: How, if it all, does patient education influence patient-centered care?
8. Participating in a study like this might have heightened your awareness about the disablement model and patient-centered care. Is there anything you think you will take with you or change in your practice as a result of participating?
9. Is there anything else you think we need to know about patient-centered care and use of the disablement model in athletic training practice?

**Table 3:** Frequency of coded categories.

Domains and Category	Frequency (*n* = 13)	Commonality
Patient-Centered Care		
Expressed Desire	84% (*n* = 11)	Typical
Conscious Competence	84% (*n* = 11)	Typical
Conscious Incompetence	38% (*n* = 5)	Variant
Unconscious Incompetence	69% (*n* = 9)	Typical
Limitations and Impairments		
Expressed Desire	15% (*n* = 2)	Variant
Conscious Competence	77% (*n* = 10)	Typical
Conscious Incompetence	0% (*n* = 0)	None
Unconscious Incompetence	31% (*n* = 4)	Variant
Environment and Support		
Expressed Desire	38% (*n* = 5)	Variant
Conscious Competence	77% (*n* = 10)	Typical
Conscious Incompetence	31% (*n* = 4)	Variant
Unconscious Incompetence	54% (*n* = 7)	Typical
Exposure		
Formal	38% (*n* = 5)	Variant
Informal	54% (*n* = 7)	Typical

## Data Availability

The data presented in this study are available upon request from the corresponding author. The data are not publicly available due to IRB protections.
